# Preparing Emergency Medicine Residents to Lead Level 1 Multidisciplinary Trauma Teams: A Scoping Review

**DOI:** 10.1002/aet2.70185

**Published:** 2026-05-14

**Authors:** Aliya S. Khan, Caroline J. Cushman, Cameran Mecham, Stephanie Stroever, Trey Morris

**Affiliations:** ^1^ School of Medicine Texas Tech University Health Sciences Center Lubbock Texas USA; ^2^ Department of Emergency Medicine Texas Tech University Health Sciences Center Lubbock Texas USA

**Keywords:** emergency medicine, level 1 trauma, residency programs, simulation‐based education, trauma leadership

## Abstract

**Background:**

In the United States, the initial management of trauma patients is coordinated by multidisciplinary teams, typically led by surgeons. As a result, Emergency Medicine (EM) residents often have limited experience in the trauma team lead role. However, in rural or critical access hospitals, EM physicians frequently serve as team leaders without surgical backup.

**Objective:**

To map and summarize educational strategies used to train EM residents to lead Level 1 trauma resuscitations and describe how outcomes such as confidence, performance, and preparedness are assessed.

**Methods:**

Following Joanna Briggs Institute scoping review methodology, a systematic search of PubMed and Embase was conducted. Inclusion criteria focused on training EM residents in trauma leadership using keywords related to education, competence, and leadership. Covidence was used for blinded screening and data extraction, and 23 studies were included following double‐blind review.

**Results:**

Simulation‐based training dominated the literature, including high‐fidelity mannequins, cadaver labs, animal models, and standardized patients. Combined approaches integrating didactics, video‐assisted debriefs, and mental rehearsal improved trauma leadership, procedural, and nontechnical skills. Most studies evaluated outcomes using self‐reported confidence and perceived competence, while fewer used validated performance assessment tools in simulated settings, and only one study evaluated resident leadership in real‐world trauma activations. Studies also assessed psychosocial constructs such as confidence and stress.

**Conclusion:**

Existing literature describes simulation as the most frequently used modality for trauma leadership training among EM residents, often paired with structured debriefing, didactics, mental rehearsal, and progressive complexity. Most studies assess learner‐reported outcomes and simulated performance, while real‐world clinical leadership assessment remains limited. Future efforts should prioritize curriculum development, standardized evaluation tools (Kirkpatrick or milestone‐based outcomes), and longitudinal models that combine simulation with real‐world practice to ensure consistent, high‐quality trauma leadership preparation.

## 1 Introduction

Emergency Medicine (EM) physicians play a crucial role in the initial evaluation and resuscitation of trauma patients, particularly in settings where surgical support is limited or delayed, such as rural or critical access hospitals. Trauma systems increasingly rely on EM clinicians for rapid decision‐making and coordination during the initial phases of care. Accordingly, the need for structured trauma leadership training during residency is increasingly emphasized. EM residency programs may lack standardized, longitudinal curricula for developing trauma leadership competencies, resulting in a wide variability in preparedness among graduating residents [1].

The trauma team leader (TTL) is responsible for guiding the resuscitation process, triaging injuries, directing diagnostic interventions, and delegating tasks across a multidisciplinary team. While the TTL does not typically perform procedures in well‐resourced medical centers, in rural or critical access facilities, the TTL may act as a proceduralist. This role requires a combination of procedural skill, cognitive flexibility, emotional regulation, and exceptional communication abilities. These skills are frequently developed through informal apprenticeship models, which may not provide uniform opportunities for all learners. In many academic and urban settings, trauma activations are led by surgical residents or fellows, limiting EM resident exposure to TTL roles during high‐acuity cases. Proposed changes to program requirements by the Accreditation Council for Graduate Medical Education (ACGME) require EM residents to serve as primary decision makers within multidisciplinary teams [2]. This may add to the challenges in obtaining experiences for both EM and surgery residents at lower‐volume trauma centers, further highlighting the need for evidence‐based curricula that improve resident competency in the role of TTL.

Simulation‐based training has emerged as a cornerstone of trauma education, offering a risk‐free environment where EM residents can practice functioning in the role of TTL, receive feedback, and build confidence [3]. High‐fidelity simulations using mannequins, cadavers, or live tissue models allow for the replication of complex trauma scenarios [3, 4, 5]. The benefits of simulation learning are well‐documented, however the optimal configuration of trauma leadership curricula remains unclear. This includes the optimal timing (PGY‐1, PGY‐2, etc.), frequency, format, and feedback mechanisms of curricular design.

Furthermore, emerging evidence suggests that nontechnical skills (NTS), such as situational awareness, closed‐loop communication, and team coordination, are as vital as procedural competence while acting in the role of TTL. Despite their importance, many residency programs do not formally assess or train for NTS, creating a critical gap in trauma education and EM training as a whole [1, 6, 7]. Additionally, prior reviews have explored resuscitation simulation and procedural trauma training; however, to the authors' knowledge, no prior scoping review has specifically mapped educational strategies and outcome assessment approaches for EM residents in the TTL role.

Given the breadth of potential training modalities and variability in programmatic design, we conducted a scoping review to map the current landscape of educational strategies used to prepare EM residents for Level 1 trauma team leadership. Our goals were to identify the most common training methods, assess their reported outcomes, and highlight opportunities for future research and standardization.

This review aligns with ACGME Milestone domains, emphasizing leadership in team‐based crisis management (Patient care 5, ICS 3).

## 2 Review Question

What educational strategies have been described to train EM residents to lead Level 1 trauma teams, and how have training outcomes (self‐reported confidence, simulated performance, or clinical leadership behaviors) been assessed?

## 3 Methods

This scoping review was conducted following the Joanna Briggs Institute (JBI) methodology for scoping reviews [8]. Our process included a rigorous and systematic approach to identify, screen, and analyze existing literature evaluating trauma leadership training for EM residents. Per JBI methodology, a formal risk of bias assessment was not performed as a scoping review intends to map existing evidence and identify gaps in knowledge [8]. The methodology was aligned with the Preferred Reporting Items for Systematic Reviews and Meta‐Analyses extension for Scoping Reviews (PRISMA‐ScR) guidelines.

The search was conducted from inception of databases to March 27, 2024. An interim literature search was performed on July 17, 2025, which did not reveal any additional qualifying articles.

### 3.1 Inclusion Criteria

Studies were eligible if EM residents participated in trauma leadership training designed to prepare residents to function as TTLs in Level 1 trauma activations or equivalent high‐acuity trauma resuscitation scenarios. Training could occur in simulation centers, hospital environments, or educational settings, provided the intervention targeted trauma leadership behaviors and decision‐making. Eligible studies described or evaluated educational interventions aimed at preparing EM residents to act as TTL, including simulation‐based training, didactic teaching, mental rehearsal, team‐based learning, case‐based discussions, and real‐world supervised exposure. Studies were included regardless of country of origin to incorporate diverse educational approaches despite differences in trauma team structures, although only English‐language publications were eligible. A variety of academic and clinical environments were considered, including large urban academic centers, suburban medical centers, and rural clinics, recognizing that trauma care structures and EM resident trauma leadership opportunities vary significantly. Accepted study designs included prospective and retrospective cohort studies, case–control studies, mixed‐methods studies, cross‐sectional analyses, and qualitative research exploring resident experiences with trauma training. Excluded study designs included systematic and scoping reviews, editorials, commentaries, and abstracts.

### 3.2 Search Strategy

A comprehensive search strategy was developed in consultation with a medical librarian. An initial search of PubMed and Embase was conducted on March 27, 2024. Keywords and controlled vocabulary terms included: “emergency medicine,” “residency training,” “trauma team leader,” “simulation,” “non‐technical skills,” “clinical competence,” and “training methods.” Search strategies were tailored to each database and refined using text word variations and MeSH/Emtree terms. Search strategies were tailored due to database‐specific indexing terms, but there were no differences in the search criteria.

**FIGURE 1:**
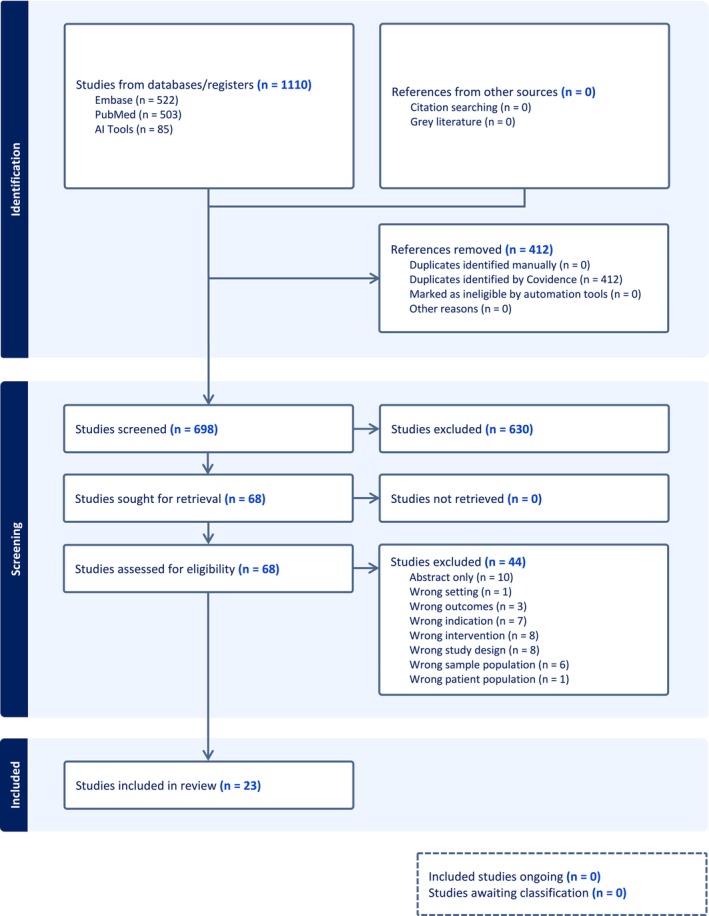
Prisma flow diagram.

The final search retrieved 914 citations. These were uploaded to Covidence, and duplicates (*n* = 314) were removed automatically. Title and abstract screening was conducted independently by two reviewers, both 3rd‐year medical students, leading to the retrieval of 59 full‐text articles. After applying inclusion and exclusion criteria, 23 studies were included (Figure 1). Reasons for exclusion included wrong population (nonresidents), wrong intervention (not related to trauma resuscitation or leadership), and wrong study design (no outcome assessment or unrelated to leadership skills).

### 3.3 Data Extraction

Two reviewers independently extracted data using a pilot‐tested form within Covidence. Discrepancies between the two primary reviewers were resolved through discussion; if consensus could not be reached, a third reviewer adjudicated the decision. Extracted information included author, year, study design, sample size, setting, training modality, measured outcomes, and conclusions. During data extraction, outcomes were categorized by assessment type to improve interpretability (learner‐reported outcomes, objective simulated performance outcomes, and clinical‐setting leadership behaviors when available), consistent with educational evaluation frameworks such as Kirkpatrick.

Although many nontechnical leadership skills overlap with those used in other acute care leadership roles, such as cardiac arrest or acute stroke resuscitation, trauma leadership was examined independently in this review due to its variability between institutional models. Although it is not clear to what extent the skill set needed for trauma resuscitations differs from that needed for medical resuscitations, current program requirements specify targets for each resuscitation type and may indicate that the skills are not identical.

## 4 Results

### 4.1 Overview of Studies

A total of 600 records were identified; 59 articles were reviewed in full, and 23 met inclusion criteria. Most studies were conducted in the United States (*n* = 19), with others from Canada (*n* = 3) and India (*n* = 1). All studies used a prospective design: pre–post surveys (*n* = 11), observational studies (*n* = 3), randomized controlled trials (*n* = 2), cross‐sectional surveys (*n* = 4), and mixed‐methods studies (*n* = 3). Sample sizes ranged from 10 to 154 participants. Study durations varied widely, from 1‐day boot camps to multimonth curricula (Table 1). A total of 980 residents (both EM and other specialties) were included.

**TABLE 1:** Summary of trauma leadership training interventions.

References, country	Study design (sample size)^a^	Measured outcomes	Model(s) of education										
			Structure of training				Simulation modality				Method of feedback		
			Simulation‐based training	Multimodal	Single session	Multiple session	Mannequins	Human patient simulation	Animal model	Cadaver model	Video debrief	Debrief	Self‐reflection
Marshall et al. [9], US	Pre–post study (*n* = 12)	Procedural competence, leadership skills	+	+	+		+					+	
Grant et al. [10], CA	Observational study (*n* = 30)	Psychological resilience, nontechnical skills, leadership skills	+	+	+		+					+	
Fernandez et al. [11], US	Randomized controlled trial (*n* = 60)	Psychological resilience, nontechnical skills, leadership skills	+	+	+		+					+	
Bonjour et al. [12], US	Observational study (*n* = 22)	Procedural competence	+	+	+		+					+	
Khobrani et al. [13], US	Survey, pre–post study (*n* = 13)	Procedural competence, nontechnical skills, leadership skills	+		+		+					+	
Hamilton et al. [14], US	Survey, pre–post study (*n* = 11)	Psychological resilience, nontechnical skills, leadership skills	+	+	+		+				+		
Wittels et al. [15], US	Cross‐sectional study, survey (*n* = 54)	Leadership skills	+			+	+					+	
Berg et al. [16], US	Survey, pre–post study (*n* = 61)	Procedural competence	+		+				+			+	
Gregg et al. [17], US	Pre–post study (*n* = 20)	Psychological resilience, nontechnical skills, leadership skills				+		+			+		
Murray et al. [18], US	Observational (*n* = 83)	Leadership skills, procedural competence	+		+		+				+		
Esparaz et al. [5], US	Survey, pre–post study (*n* = 50)	Procedural competence	+		+					+		+	
Dagnone et al. [19], CA	Pre–post study (*n* = 30)	Procedural competence, nontechnical skills, leadership skills	+	+		+	+					+	
Onufer et al. [3], US	Survey, Pre post study (*n* = 119)	Procedural competence, nontechnical skills	+		+				+	+		+	
Kelley et al. [20], US	Survey (*n* = 18)	Procedural competence, nontechnical skills, leadership skills	+		+		+					+	
Lorello et al. [7], CA	Randomized controlled trial (*n* = 78)	Psychological resilience, nontechnical skills, leadership skills	+		+		+					+	
Caldwell et al. [21], US	Survey, pre–post study (*n* = 18)	Procedural competence, leadership skills	+	+	+		+					+	
Onufer et al. [22], US	Survey, pre–post study (*n* = 79)	Leadership skills	+		+		+					+	
Huffman et al. [23], US	Pre–post study (*n* = 25)	Nontechnical skills	+		+		+					+	
Anton et al. [24], US	Survey, pre–post study (*n* = 49)	Psychological resilience, nontechnical skills, leadership skills	+	+		+	+					+	+
Rosenman et al. [6], US	Survey, observational study (*n* = 36)	Leadership skills	+	+	+		+					+	
Anton et al. [25], US	Survey, Pre–post study (*n* = 41)	Psychological resilience, nontechnical skills, leadership skills	+			+	+					+	+
Jogerst et al. [26], US	Pre–post study (*n* = 79)	Procedural competence, nontechnical skills	+		+		+				+		
Gartland et al. [27], US	Pre–post study (*n* = 24)	Psychological resilience, nontechnical skills, leadership skills	+	+	+		+					+	

Abbreviations: CA, Canada; IN, India; US, United States.

^a^ The sample size listed is the total number of residents included in each study.

### 4.2 Simulation‐Based Training

Across all studies, high‐fidelity simulation was the most widely used training modality (*n* = 23). Among these, many utilized mannequins (*n* = 20) to replicate common trauma scenarios, and few used cadaveric models (*n* = 2) and live animal models (*n* = 2) to create artificial trauma scenarios. The training occurred in both dedicated simulation labs and hospital environments, both of which were tailored to reflect actual clinical environments in which EM residents would lead trauma resuscitations.

In several studies, researchers introduced multidisciplinary collaboration by involving residents from multiple specialties in simulation‐based trainings in order to accurately model the environment of Level 1 trauma activations [21]. Furthermore, many studies aimed to replicate real‐world trauma situations by incorporating distractions, giving residents limited information, and having patients present with evolving symptoms. Residents were required to perform assessments, delegate tasks, communicate effectively, and adapt to rapidly changing priorities. Performance was evaluated using tools such as the Team Emergency Assessment Measure (TEAM), Clinical Teamwork Scale, Ottawa Global Rating Scale, Mayo Performance Teamwork Scale, and other institution‐specific tools which were not externally validated. Other studies evaluated the effect of simulation on response time and the efficiency of resuscitation [12].

Structured debriefing sessions were commonly incorporated into simulation‐based curricula (*n* = 20), typically focusing on leadership behaviors, communication clarity, procedural performance, and teamwork dynamics. Studies incorporating video review (*n* = 4) and self‐reflection (*n* = 2) components reported improvements in learner‐reported retention and leadership behaviors within simulated scenarios. Structured workshops that included explicit leadership instruction frequently reported increases in leadership‐related self‐efficacy following the intervention [10].

Several studies reported using simulation as a single‐session intervention (*n* = 17), while others employed repeated or longitudinal exposure (*n* = 6). Across the studies, many reported that even a single simulation session could significantly improve resident confidence in managing trauma teams. Boot camps can serve as a scalable model for these interventions [13]. Longitudinal curricula provided repeated leadership exposure across training periods and commonly reported improvements in confidence and simulated leadership performance over time [27].

### 4.3 Multimodal and Tiered Curricula

Ten studies incorporated a multimodal or tiered structure that blended simulation with additional educational components. Among these, seven combined simulation with didactic lectures, five incorporated structured video playback, four used mental rehearsal modules, and three included team‐based learning exercises.

Across multimodal curricula (*n* = 10), studies most frequently reported improvements in learner satisfaction, confidence, and simulated leadership performance following participation. Several studies also described enhanced performance during high‐stress simulation scenarios, although outcome measures varied substantially across studies.

### 4.4 Real‐World Exposure and Practice

Current literature underlines the critical nature of real‐world exposure plays in harnessing and adapting the lessons learned during simulation‐based training into clinical practice [17]. Despite these findings, opportunities for EM residents to gain experience in TTL‐role remain limited; in this review, only one study (Gregg et al.) offered a real‐world training opportunity [17]. However, one retrospective study evaluated real‐world trauma leadership performance and found that EM residents achieved TTL outcomes comparable to surgical residents during supervised trauma resuscitations. Leadership performance was assessed using an institution‐specific evaluation tool, with no significant differences found between groups [28].

While academic centers may offer high trauma volumes, they also often restrict EM resident autonomy. Conversely, rural programs offer more autonomy to EM residents with more opportunities in real‐world exposure and the possibility of assuming the TTL role due to the absence of surgical coverage while they may lack critical feedback from traumatologists found at academic training sites. The lack of standardized teaching protocols or faculty support calls into question the efficacy of these potential opportunities for resident learners [29].

In Gregg et al., the authors implemented structured, supervised real‐world trauma leadership opportunities. This study was performed at a Level 1 Trauma center on a sample size of 20 residents with resident exposure varying in levels of traumatic acuity [17]. In this model, a junior resident performed as team lead in a real‐time trauma scenario while being evaluated by a senior resident [17]. The junior resident then received feedback from an evaluation tool developed by the authors which involved a 30‐item, ACGME‐competency‐based Likert scoring framework. This method leverages real clinical experiences to enhance learning. Additionally, the personalized feedback sessions were a key strength. These sessions allowed residents to reflect on their performance right after the event, which can reinforce learning and foster continuous improvement.

Although the training model has produced promising initial results for those involved, limitations and barriers beyond those previously listed in this section limit the broad implementation of the outlined protocol. Possible concerns are patient safety, institutional liability, attending oversight requirements, and coordination with consulting services represent important hurdles to adoption. Logistical hurdles, such as uneven resident exposure to trauma cases in total number as well as acuity level, also represent a potential risk in reproducibility among programs and should be weighed appropriately in future studies. Despite the logistical and institutional hurdles outlined, there remains an opportunity for real‐world exposure and high fidelity learning for EM residents through the implementation of protocols such as Gregg et al. outlined. This data suggests a need for greater institutional cooperation to support training programs, academic or community or otherwise, in their effort to prioritize EM resident real‐world exposure in leading traumas.

The implementation of techniques and teaching strategies outlined in the previous sections is practiced and implemented in artificial environments through imitation‐based practices in the effort of replicating real‐world scenarios to correctly officiate in the TTL role. The opportunity for EM residents to assume the TTL role to run trauma activations under supervision with planned feedback represents a potential opportunity for each resident to translate learned knowledge into clinical competency.

### 4.5 Measured Outcomes

Across included studies, outcomes were primarily assessed using learner‐reported measures such as confidence, perceived competence, preparedness, self‐efficacy, satisfaction, stress, and anxiety, and simulated performance‐based assessments, while clinical‐setting leadership assessment was rare. Measured outcomes were grouped into four domains: leadership skills, NTS, procedural competence, and psychological resilience.

Leadership skills were the most frequently assessed outcome and were reported in 18 studies using structured tools and simulation performance metrics [27]. Some studies (*n* = 9) employed validated assessment tools such as the TEAM or Ottawa Global Rating Scale [10]. These studies consistently demonstrated significant improvements in communication clarity, prioritization, delegation of tasks, and overall team direction following intervention. Many studies (*n* = 14) employed institution‐specific, nonvalidated tools to assess trauma leadership performance. Notably, TTL effectiveness was enhanced in programs that incorporated video debriefings and structured feedback mechanisms, which allowed residents to critically reflect on their actions and receive targeted guidance [20].

NTS, a pivotal component of trauma team leadership, were evaluated in 16 studies. These skills included closed‐loop communication, situational awareness, assertiveness, and coordination under pressure. Programs integrating cognitive rehearsal, peer evaluation, crisis resource management, or interprofessional simulation exercises demonstrated enhanced teamwork performance [23, 26]. For example, Onufer et al. revealed improved communication and decision‐making among participants exposed to multimodal training [22]. The inclusion of NTS training is critical, as it addresses human factors that significantly influence patient safety during high‐acuity resuscitations [30].

Procedural competence was evaluated in 12 studies, often focusing on technical interventions such as airway management, hemorrhage control, and chest decompression. Interventions that utilized cadaver or animal models were found to significantly increase resident comfort and success in performing rare, high‐stakes procedures [5, 18]. The effect of cadaver‐based simulation is well‐documented in the surgical setting; in many studies, structured cadaver surgical training programs have been shown to produce significant gains in procedural competence and confidence [31]. Procedural skills are vital for TTLs, especially in settings where procedural support may be limited or delayed.

Psychological resilience and stress response were reported in eight studies. These studies examined both subjective perceptions of stress and objective measures such as cortisol sampling. Anton et al. incorporated mindfulness components designed to decrease physiologic stress markers into high‐fidelity trauma simulation. This study showed that stress has a negative effect on NTS during trauma simulation [25]. This is especially relevant, as the TTL must make critical decisions while managing personal and team anxiety in chaotic circumstances (Table 2).

**TABLE 2:** Classification of outcome measures across included trauma team leadership training studies (self‐reported, objective simulation‐based, and objective clinical outcomes).

Study	Subjective/self‐report	Objective simulation with tool/metric	Objective clinical—with tool/metric
Marshall et al. [9]	NR	Procedural/management performance using human patient simulator	Did not include
Grant et al. [10]	Did not include	Objective simulated leadership/competence using a developed evaluation tool	Did not include
Fernandez et al. [11]	Perceived leadership/nontechnical skill improvement (participant ratings)	Structured ratings of leadership performance in simulation	Yes – leadership performance during actual trauma resuscitations
Ahluwalia et al. [32]	Confidence/perceived competence survey (pre/post)	Procedural/nontechnical performance evaluation in sim (tool not specified)	Did not include
Bonjour et al. [12]	NR	Objective resuscitation/evaluation time metrics in simulation	Did not include
Khobrani et al. [13]	Confidence/comfort/preparedness survey (pre/post boot camp)	Simulated procedural/leadership performance	Did not include
Hamilton et al. [14]	Did not include	Objective team function using video review + reliable evaluation metric	Did not include
Wittels et al. [15]	Resident curriculum evaluation/satisfaction survey	NR	Did not include
Berg et al. [16]	Perceived procedural competence/confidence survey	Procedural competence assessment in simulation	Did not include
Gregg et al. [17]	NR	Objective leadership behaviors using real‐time competency‐based evaluation tool	Did not include
Murray et al. [18]	NR	Objective diagnostic/decision‐making performance in simulation	Did not include
Esparaz et al. [5]	NR	Objective invasive procedure performance in simulation	Did not include
Dagnone et al. [19]	Learner‐perceived value/confidence with resuscitation training	Objective simulation performance outcomes	Did not include
Onufer et al. [3]	Learner perceptions of preparedness/comfort with trauma technical skills and management exposure	Objective simulation‐based technical skills assessment/exposure	Did not include
Kelley et al. [20]	Confidence/comfort performing surveys + leadership preparedness	Objective simulated survey performance assessment	Did not include
Lorello et al. [7]	NR	Objective performance in team trauma simulation after mental practice	Did not include
Caldwell et al. [21]	Self‐reported confidence/preparedness leading/evaluating trauma resuscitation	Objective multidisciplinary team performance in simulation	Did not include
Onufer et al. [22]	Self‐reported confidence/preparedness + perceived competency gaps	Objective simulation‐based performance evaluation	Did not include
Huffman et al. [23]	NR	Objective nontechnical skills/crisis resource management performance ratings	Did not include
Anton et al. [24]	Self‐reported stress/resilience and/or leadership confidence survey	Objective simulation performance	Did not include
Rosenman et al. [6]	Self‐reported leadership confidence/comfort leading trauma resuscitation	Objective leadership ratings in simulation	Did not include
Anton et al. [25]	Stress ratings and perceptions of interdisciplinary teamwork	Objective team performance ratings in trauma simulation	Did not include
Jogerst et al. [26]	Perceived competence/confidence with procedural skill performance	Objective procedural performance in interprofessional simulation	Did not include
Gartland et al. [27]	Learner self‐assessment/confidence with resuscitation leadership	Objective simulation performance metrics/ratings	Did not include

## 5 Discussion

This scoping review summarizes trauma leadership training strategies for EM residents. Simulation‐based education was the most frequently reported approach and was often paired with structured debriefing, didactics, and mental rehearsal. While simulation was the most leveraged modality, it achieves maximal impact when paired with structured debriefing, didactics, and mental rehearsal. Programs incorporating video‐assisted feedback or team‐based approaches frequently reported improvements in learner confidence and simulated leadership performance [14, 20]. These integrated approaches aimed to target both the cognitive and behavioral aspects of trauma leadership development.

A central theme across studies is the vital importance of NTS development. Trauma team leadership is not solely procedural; it demands the ability to communicate effectively, manage resources, and lead under uncertainty. Studies by Lorello et al. and Onufer et al. underscore how mental rehearsal and peer evaluation can meaningfully improve communication and situational awareness, skills indispensable to trauma team performance [3, 7, 22]. Other studies highlight the importance of interprofessional collaboration development to lead a multidisciplinary team [26]. Cognitive flexibility is another essential skill for trauma leads; studies show that simulated trauma care improves real‐time decision making [18].

Beyond preference, structural and institutional factors influence how trauma team leadership training is delivered within EM residency programs. The review presented demonstrates that TTL is variably addressed in EM residency training and when targeted often relies on simulation. EM residency programs face numerous structural barriers when implementing TTL training for EM residents, including addressing institutional policies and coordinating with other specialties such as surgery who also require TTL experience. Co‐located EM and surgery residencies at trauma sites with lower volumes of Level 1 traumas and programs with relatively larger numbers of residents for their trauma volume may face resistance from surgery PDs looking to maximize their own residents' experience. Increased EM resident exposure to rural or lower‐resourced sites which are less likely to have surgery residents in‐house may improve hands‐on experience and is supported by the proposed program requirements under consideration by the EM RRC.

An alternative approach might be to increase EM resident exposure to critical care settings. However, it is unknown to what extent medical resuscitation team lead experience transfers to TTL performance. The applicability of resuscitation experience in the ICU is likely limited given the ED's central role in initial stabilization for many patients.

Real‐world validation of trauma leadership training remains limited but essential. Most studies measured performance using simulation‐based checklists or subjective self‐assessments. Gregg et al. are notable for implementing a real‐world exposure, but scalability is challenged by ethical, legal, and logistic constraints [17]. To translate training into patient outcomes, future research should pursue objective measures, such as time to intervention, adherence to trauma protocols, or patient morbidity and mortality.

Butler, et al. represents an important counterpoint to concerns regarding EM residents' preparedness to serve as TTL; it demonstrated that within their sample, EM residents performed equally well as surgical residents at leadership performance [28]. This finding suggests that, under certain training conditions, EM residents are capable of serving as team leads. However, this study represents a single institutional experience and does not negate the broader variability which exists in exposure to Level 1 trauma among EM residents. Rather, it highlights that comparable performance is achievable when structured opportunities and supervision are in place [28]. The limited number of studies which evaluate real‐world trauma leadership underscores the need for more standardized and generalizable approaches to trauma leadership training.

Importantly, EM residency is a progressive learning environment, and training must reflect this. Junior residents benefit most from structured environments rich in feedback, while senior residents thrive in autonomous, complex scenarios [28]. A tiered, longitudinal training model could progressively develop trauma leadership competencies across PGY levels, as shown in Fernandez et al.'s tiered feedback system [11].

To enhance trauma leadership preparation, programs should implement a combination of recurring high‐fidelity simulation, formal instruction on NTS, and guided self‐assessment through video debriefing. Structured mental rehearsal protocols and trauma leadership checklists can be introduced early in residency and reinforced longitudinally. Programs should also consider creating institutional policies that delineate EM resident leadership roles in trauma settings and provide real‐time, supervised opportunities to lead Level 1 trauma activations. Some studies incorporated wellness‐oriented components such as mindfulness‐based strategies alongside simulation. Future curricula may consider integrating evidence‐informed approaches to stress modulation and emotional regulation during high‐acuity events, as integrating stress inoculation training and emotional regulation modules may further strengthen resident performance in chaotic, high‐stakes settings [22]. Leadership behaviors in the included studies were often framed narrowly, with assertiveness used the most frequently [10, 14]. This highlights an opportunity for future work to incorporate a multidimensional view of leadership.

Another major limitation in the literature is the lack of standardization. Outcome measures varied widely, and validated leadership assessment tools were rarely used. While Emergency Stabilization is a core EM milestone domain, explicit language describing leadership of trauma resuscitations is not consistently represented, emphasizing the need for clearer expectations and assessment approaches for trauma team leadership skills. This heterogeneity hinders interstudy comparison and generalizability. Future work may benefit from linking trauma leadership assessment to established graduate medical education frameworks, including milestone‐based behavioral anchors and entrustable professional activities, to improve consistency and benchmarking across programs. For instance, the EM PC1 (“Emergency Stabilization”) milestone does not explicitly include trauma or resuscitation leadership language, whereas the equivalent Pediatric EM PC6 milestone incorporates leadership expectations during emergency stabilization [33].

Psychosocial preparedness is another underexplored yet critical area. The role of TTL during trauma resuscitation is psychologically demanding. Anton et al. showed that stress levels can affect communication and decision‐making, and interventions that target resilience may enhance performance under pressure [25]. However, stress‐inoculation interventions are not universally perceived as a viable wellness strategy, and may be perceived by some learners as punitive. Mindfulness strategies may serve as a viable alternative to provide residents with tools to self‐regulate in chaotic environments [15]. Despite its importance, few curricula included elements of psychosocial preparedness, suggesting an opportunity to integrate wellness into performance training.

Finally, national consensus on trauma leadership training within EM is lacking. Without a standardized curriculum, residents' experiences and preparedness remain variable [1, 28, 29]. Establishing national guidelines or competencies, such as milestone‐based trauma leadership expectations across PGY levels could enhance equity and consistency, particularly in low‐resource or community‐based training sites where surgical support may be inconsistent. These structured curricula may enhance skill retention and confidence [9]. Additionally, funding and faculty development are critical to sustain these interventions, particularly for smaller or rural programs with limited infrastructure. This synthesis provides programs with an evidence‐informed array of curricular strategies and outcome measures which can be adapted to various institutions.

The authors did not identify studies evaluating virtual or augmented reality‐based interventions for trauma leadership training in EM residents, representing a potential future direction for curriculum development and evaluation. Future studies should explore the feasibility and scalability of integrated trauma leadership curricula across varied training environments and assess the long‐term impact on patient outcomes and clinical performance postresidency.

### 5.1 Limitations

This scoping review has several limitations. First, studies were heterogeneous in setting, intervention design, and outcome measurement, limiting comparability across curricula. Second, most studies relied on learner‐reported outcomes and simulation‐based assessments, with limited evaluation of clinical leadership performance or patient‐level outcomes. Third, educational interventions may be underrepresented in the peer‐reviewed literature, as many residency curricula are implemented locally without publication. Finally, only English‐language publications were included, which may exclude relevant international work.

### 5.2 Implications for EM

These findings can inform the ACGME Milestone mapping and structured trauma leadership curricula across EM residency programs. Programs may integrate these findings into current resident assessment frameworks.

## 6 Conclusion

This scoping review affirms the value of integrating high‐fidelity simulation, multimodal instruction, and supervised clinical exposure in trauma leadership training for EM residents. Simulation‐based education serves as a foundational pillar, enabling experiential learning in controlled, high‐stakes environments. However, simulation‐based training may be strengthened when paired with structured feedback, mental rehearsal, and progressive complexity, as seen in tiered curricula. Real‐world experience, though underutilized in literature, could be a powerful complement to simulation, translating classroom gains into clinical confidence and competency and represents a potential area for future curriculum evaluation when feasible. Programs should strive to overcome institutional barriers and create opportunities for EM residents to assume leadership roles during trauma resuscitations under supervision. NTS and stress resilience are integral to trauma leadership yet remain underrepresented in training models. Incorporating validated tools for assessing communication, teamwork, and emotional regulation is essential for developing competent trauma leaders. Future directions include establishing national guidelines for trauma leadership training within EM, creating validated assessment instruments, and expanding longitudinal programs that combine simulation with real‐world exposure. Standardization across residency programs will help ensure that all EM graduates, regardless of training environment, are equipped to lead trauma resuscitations safely and effectively.

## Author Contributions

**Trey Morris:** conceptualization, supervision, writing – review and editing. **Aliya S. Khan:** investigation, formal analysis, data curation, writing – original draft, writing – review and editing, visualization. **Caroline J. Cushman:** investigation, writing – original draft, visualization, writing – review and editing, formal analysis, data curation. **Cameran Mecham:** conceptualization, writing – review and editing. **Stephanie Stroever:** methodology, validation, formal analysis, writing – review and editing, visualization, supervision, project administration.

## Funding

The authors have nothing to report.

## Conflicts of Interest

The authors declare no conflicts of interest.

## Data Availability

The data that support the findings of this study are available from the corresponding author upon reasonable request.
